# Assessing the effectiveness of environmental sampling for surveillance of foot-and-mouth disease virus in a cattle herd

**DOI:** 10.3389/fvets.2023.1074264

**Published:** 2023-03-13

**Authors:** John Ellis, Emma Brown, Claire Colenutt, Simon Gubbins

**Affiliations:** Transmission Biology, Pirbright Institute, Surrey, United Kingdom

**Keywords:** environmental surveillance, foot-and-mouth disease, FMDV, mathematical model, cattle, epidemiology

## Abstract

The survival of foot-and-mouth disease virus (FMDV) in the environment provides an opportunity for indirect transmission, both within and between farms. However it also presents the possibility of surveillance and detection *via* environmental sampling. This study assesses the effectiveness of environmental sampling strategies in the event of an outbreak, using a previous model for transmission of FMDV in a cattle herd that had been parameterized using data from transmission experiments and outbreaks. We show that environmental sampling can be an effective means of detecting FMDV in a herd, but it requires multiple samples to be taken on multiple occasions. In addition, environmental sampling can potentially detect FMDV in a herd more quickly than clinical inspection. For example, taking 10 samples every 3 days results in a mean time to detection of 6 days, which is lower than the mean time to detection estimated for the 2001 UK epidemic (8 days). We also show how environmental sampling could be used in a herd considered to be at risk as an alternative to pre-emptive culling. However, because of the time taken for virus to accumulate at the start of an outbreak, a reasonable level of confidence (> 99%) that an at-risk herd is indeed free from infection is unlikely to be achieved in less than 1 week.

## 1. Introduction

Foot-and-mouth disease (FMD) is a highly infectious disease, affecting cloven-hoofed animals such as cattle, sheep, goats, pigs and various wildlife species (1). The causative agent, foot-and-mouth disease virus (FMDV), is spread primarily through direct contact between infected and susceptible animals. Indirect transmission can occur *via* the environment and long distance transmission is facilitated through fomites or aerosols. In disease-free countries, FMDV can spread rapidly upon introduction, causing significant disruption and economic costs (2). The outbreak of FMD in the UK in 2001 resulted in the culling of 4.2 million animals for disease control purposes, another 2.3 million on welfare grounds, and costs of over £8 billion (3, 4). To reduce the spread between farms and bring the outbreak under control, the time between the first infection on a farm and the reporting of infection is vital.

FMDV is shed from infected animals into their environment through their excretions and secretions, potentially remaining infectious for a prolonged period of time (depending on environmental conditions, such as temperature and humidity) (5–7). The accumulation of FMDV in the environment also provides the opportunity for environmental sampling as a means of detecting virus circulation. This has been successfully demonstrated in countries where FMD is endemic in previous studies (7, 8). Furthermore, taking environmental swabs [see, e.g., (5, 7, 8)] is a non-invasive alternative to clinical examination, requires little prior knowledge of diseases or handling of animals and is low cost in terms of sample collection.

In the event of a future outbreak of FMD in the UK or other FMD-free country, environmental sampling could alleviate some of the burden of having experienced veterinarians examine large numbers of animals and reduce the detection time of suspected cases. It could also be used to monitor at-risk farms as an alternative to pre-emptive culling, thereby reducing the number of animals culled. Because of the ability to infer the disease status of a population without testing many individuals, environmental surveillance has also been utilized for other pathogens that cause animal diseases such as Johne's disease (9) and avian influenza (10), as well as human diseases including COVID-19 (11, 12) and polio (13–15).

Here, we assess environmental sampling as a means of FMDV surveillance for a single herd. We use an individual based model of FMDV transmission within a cattle herd where infection of a susceptible individual can occur through direct contact with an infected animal or through environmental contamination (16). The model estimates the amount of FMDV that accumulates in the environment where a herd is located as an outbreak develops. Using this, we estimate the probability of detecting FMDV in an environmental sample at any given moment during the outbreak. Different surveillance strategies, which vary in the number of samples taken and time intervals between sampling, are considered and the time from infection to detection is calculated. We also assess the utilization of environmental surveillance in a herd at risk of infection as an alternative to pre-emptive culling.

## 2. Methods

### 2.1. Transmission model

We have previously developed an individual based model for the within-herd transmission of FMDV that includes transmission *via* direct contact and *via* a contaminated environment (16).

#### 2.1.1. Viral shedding

Following infection, the infectiousness of an animal is proportional to the level of viral shedding. The level of virus in an infected animal can be modeled as

(1) $$\begin{array}{l} V\left(\tau \right)=\frac{2V_{p}}{\exp\left(-\gamma_{g}\left(\tau -T_{p}\right)\right)+\exp\left(\gamma_{d}\left(\tau -T_{p}\right)\right)}, \end{array}$$

where *V*_*p*_ is the level of peak titre, *T*_*p*_ is the time of peak titre, γ_*g*_ and γ_*d*_ are the rates for the exponential viral growth and decay phases, respectively, and τ is the time since infection. The corresponding level of viral shedding is given by

(2) $$\begin{array}{l} S\left(\tau \right)=\log\left(V\left(\tau \right)\right) \end{array}$$

where *V*(τ) is given by Equation (1) and *S*(τ) is restricted to be non-negative.

Variation amongst individuals in shedding is incorporated in the model by sampling the parameters from higher-order distributions. More specifically, γ_*g*_, γ_*d*_ are drawn from gamma distributions with means μ_γ_*g*__, μ_γ_*d*__ and shape parameters *s*_γ_*g*__, *s*_γ_*d*__, respectively. *V*_*p*_ is drawn from a log gamma distribution with parameters μ_*V*_ and *s*_*V*_ (the mean and shape parameter of the corresponding gamma distribution). Finally, the time of peak titre *T*_*p*_ and incubation period *T*_*c*_ are drawn from a bivariate log normal distribution with parameters μ_*T*_*p*__, μ_*T*_*c*__, σ_*T*_*p*__ and σ_*T*_*c*__ (the means and standard deviation of the corresponding normal distribution) and a correlation coefficient ρ_*pc*_. This allows the within-host viral dynamics to be linked to the onset of clinical disease.

#### 2.1.2. Environmental contamination

The rate of environmental contamination from each animal is assumed to depend on the amount of virus shed by an individual (given by Equation 2) and the natural decay rate of virus in the environment. The contamination and decay rates are assumed to vary between four areas: the floor, walls, trough, and feces. The level of virus in each location is given by

(3) $$\begin{array}{l} \frac{dE_{i}}{dt}=\frac{\alpha_{i}}{N}\overset{N}{\underset{j=1}{\sum }}S_{j}\left(t\right)-\delta_{i}E_{i}\left(t\right), \end{array}$$

where *E*_*i*_, *i* = 1, …, 4 is the level of contamination found in the floor, walls, trough, and feces, respectively. α_*i*_ is the rate of contamination, δ_*i*_ is the rate of decay and *N* is the herd size.

#### 2.1.3. Probability of transmission

Transmission of FMDV within the herd can occur through direct contact between animals or through environmental contamination. For direct transmission, the probability of an animal becoming infected through direct transmission over a time interval [*t, t* + Δ*t*] is given by

(4) $$\begin{array}{l} P_{d}\left(t\right)=1-\exp\left(-\beta_{d}\int_{t}^{t+\Delta t}\frac{\sum_{j=1}^{N}S_{j}\left(t\right)}{N}dt\right), \end{array}$$

where β_*d*_ is the direct transmission rate. The probability of a susceptible animal becoming infected *via* environmental contamination in the interval [*t, t* + Δ*t*] is given by

(5) $$\begin{array}{l} P_{e}\left(t\right)=1-\exp\left(-\beta_{e}\int_{t}^{t+\Delta t}\overset{4}{\underset{i=1}{\sum }}E_{i}\left(t\right)dt\right), \end{array}$$

where β_*e*_ is the environmental transmission rate which is assumed to be the same for all contaminated areas. The probability for a susceptible animal to become infected at each time interval is given by

(6) $$\begin{array}{l} P\left(t\right)=\left(1-\left(1-P_{e}\left(t\right)\right)\left(1-P_{d}\left(t\right)\right)\right). \end{array}$$

**Figure 1:**
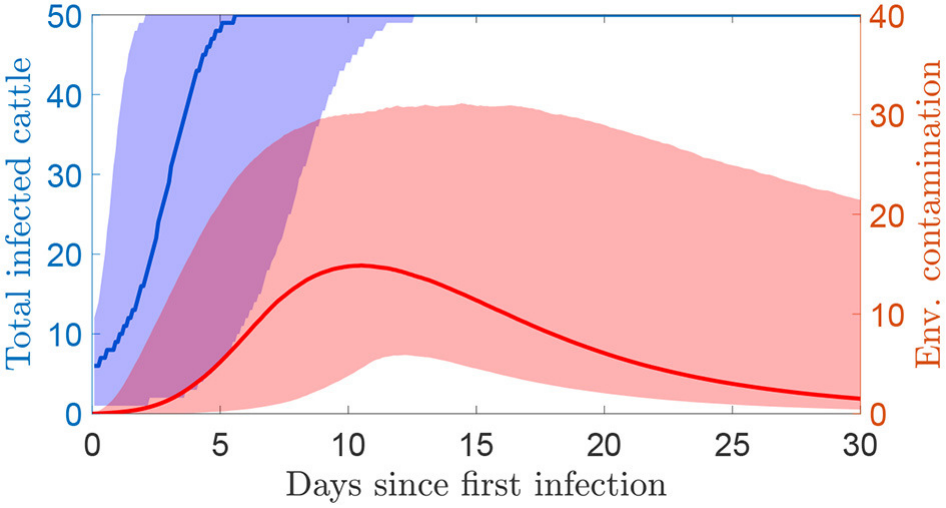
The simulated number of infected cattle and the total environmental contamination over time since the first infections using the transmission model (median and 95% prediction interval). Environmental contamination is taken as the sum of the measures at each of the four locations. The herd size is 50 cattle.

The dynamics of a within-herd outbreak described by this model are discussed in more detail in (16). An example of an outbreak in terms of the number of infected cattle and total environmental contamination over time is shown in Figure 1. The level of environmental contamination is used to estimate detection times.

### 2.2. Detection of FMDV in environmental samples

We assume environmental samples will be tested by an rRT-PCR assay specific for FMDV (6, 7). The probability of detecting FMDV in an environmental sample depends on the amount of virus in the location (i.e., walls, floor, feed trough, or feces) being sampled. We assume the probability is given by

(7) $$\begin{array}{l} P\left(E_{j}\right)=1-\exp\left(-\xi_{j}E_{j}\right), \end{array}$$

where ξ_*j*_ is a parameter relating the level of environmental contamination to the probability of detection and *E*_*j*_, *j* = 1, …, 4 is the level of environmental contamination on the walls, floor, trough, or feces, respectively. This probability incorporates the sensitivity of the test as well as the chances of finding virus in the location sampled. The test specificity is assumed to be equal to one.

### 2.3. Parameter estimation

Parameters in the transmission model were estimated previously using approximate Bayesian computation sequential Monte Carlo (ABC-SMC) (16). In the present study, a sample was drawn from the joint posterior distribution for the parameters and used in the simulation for each replicate.

The parameter ξ_*j*_ was estimated from data on the amount of virus and the proportion of positive samples from environmental samples collected during a series of transmission experiments (6). The likelihood for the data is given by,

(8) $$\begin{array}{l} L=\underset{E_{j}}{\prod }\left(\begin{array}{c} n_{E_{j}}\\ k_{E_{j}} \end{array}\right)P{\left(E_{j}\right)}^{k_{E_{j}}}{\left(1-P\left(E_{j}\right)\right)}^{n_{E_{j}}-k_{E_{j}}} \end{array}$$

where *n*_*E*_*j*__ and *k*_*E*_*j*__ are the number of samples taken and the number of positive samples at each level of estimated environmental contamination *E*_*j*_. The posterior distribution was generated using an adaptive Metropolis-Hastings algorithm with an non-informative uniform prior and a Gaussian proposal distribution, scaled to ensure an acceptance rate between 30 and 50%. For each replicate a value for ξ was drawn from its posterior distribution independently of the transmission parameters.

### 2.4. Environmental sampling strategies

Combining the transmission model, which simulates the level of environmental contamination over time, with the probability of detection, we can explore the effectiveness of different sampling strategies. For each strategy we assume that *s* samples are taken from the environment every *d* days, starting at a random day post-infection between 1 and *d*. The location of the samples is random, i.e., for each sample a number from one to four is randomly generated and the sample is taken at the corresponding area of the environment.

#### 2.4.1. Sampling to detect an infected herd

The effectiveness of a strategy at detecting an infected herd was assessed in two ways. First, we estimated the time to detection, which is the number of days after the initial infection in the herd that an environmental sample tests positive under the sampling strategy. Second, we calculated the proportion of infectiousness that occurs before detection, which is indicative of how transmission could occur to other herds before detection.

As the transmission model includes two routes of transmission, the proportion of infectiousness can be estimated for each. The proportion of infectiousness from infected cattle is given by

(9) $$\begin{array}{l} \theta_{S}=\frac{\int_{0}^{t_{d}}\sum_{j=1}^{N}S_{j}\left(t\right)dt}{\int_{0}^{\infty }\sum_{j=1}^{N}S_{j}\left(t\right)dt}, \end{array}$$

and the proportion of infectiousness from environmental contamination is given by

(10) $$\begin{array}{l} \theta_{E}=\frac{\int_{0}^{t_{d}}\sum_{i=1}^{4}E_{i}\left(t\right)dt}{\int_{0}^{\infty }\sum_{i=1}^{4}E_{i}\left(t\right)dt}, \end{array}$$

where *t*_*d*_ is the time at which detection occurs.

For a strategy to be effective it needs to reduce the between-herd basic reproduction ratio *R*_*h*_ to below one. The upper confidence limit of *R*_*h*_ for farms during the initial phase of the 2001 UK FMD epidemic was estimated to be 3.2 (17, 18). We use this figure as a conservative estimate of the *R*_*h*_ with no surveillance to demonstrate the sampling effort required to reduce transmission so that *R*_*h*_ < 1, the point at which an epidemic can not sustain itself. This requires θ < 1/*R*_*h*_ ≈ 0.31.

#### 2.4.2. Sampling in an at-risk herd

An alternative use of environmental sampling is in a herd deemed at risk of infection but in which no animals have shown clinical signs. This could be to detect infection, if it is present, as early as possible or as a means of showing the herd is free from infection as an alternative to pre-emptive culling. Sampling should begin as soon as FMDV is detected on the other farm and continue until either FMDV is detected on the at-risk farm or sufficient samples have been taken over a long enough time period to have confidence that FMDV is not present on the at-risk farm.

Given a time interval in which infection could have occurred, it is possible to show how many samples need to be taken for how many days consecutively before the probability that an infected herd would remain undetected is less than a given threshold. Detection occurs when any single sample returns a positive result. Therefore, we calculate the probability of a sampling strategy being negative as the product of negative results from all samples taken over the entire period of time when sampling is undertaken. We repeat 10,000 simulations and, starting on a random day within the given interval, test *s* samples for the next *d* days. The proportion of simulations that do not result in detection provides an estimate for the probability that all samples on an infected farm would be negative.

#### 2.4.3. Comparison with clinical surveillance

Using the results given by the model, we can simulate the time taken from infection to detection using clinical surveillance. The onset of clinical signs is included in the transmission model and so this alternative detection method can be modeled in a similar manner to taking environmental samples. At each inspection interval, a given number of animals are randomly selected for inspection and the outbreak is detected if at least one of them is showing clinical signs.

We also consider the time taken from infection to reporting for farms during the 2001 UK outbreak, which was estimated to follow a gamma distribution with a mean of 8.07 days and a variance of 6.67 (19).

## 3. Results

### 3.1. Probability of detection

**Figure 2:**
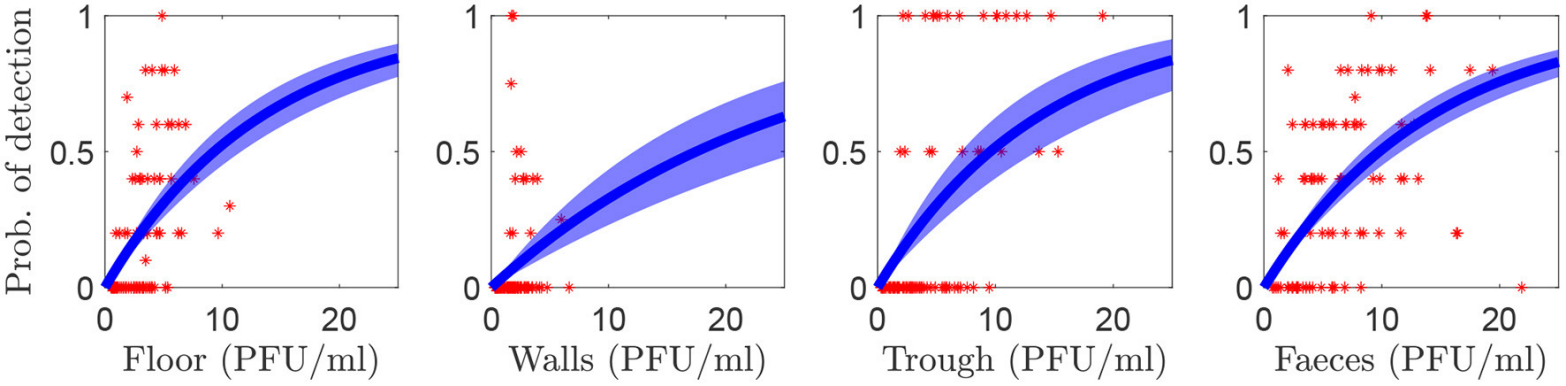
The probability of an environmental sample being positive for FMDV by rRT-PCR at different levels of environmental contamination. Red dots show the proportion of samples that were positive for FMDV when sampled at the same level of predicted environmental contamination. The blue line shows the posterior median and the shaded area shows the 95% credible interval.

The posterior median and 95% credible interval for ξ is shown in Table 1, and the probability of detection at different levels of contamination is shown in Figure 2. The results are similar for each area of the environment except for the walls, which has a lower ξ and a shallower probability curve. Note that at each contamination level only a few samples were taken, which is why the proportion testing positive appears to take discrete values.

**Table 1:** The median and 95% credible interval of ξ.

	**Floor**	**Walls**	**Food trough**	**Feces**
ξ	0.075 (0.060–0.091)	0.040 (0.026–0.057)	0.073 (0.051–0.098)	0.071 (0.060–0.083)

### 3.2. Sampling to detect an infected herd

**Figure 3:**
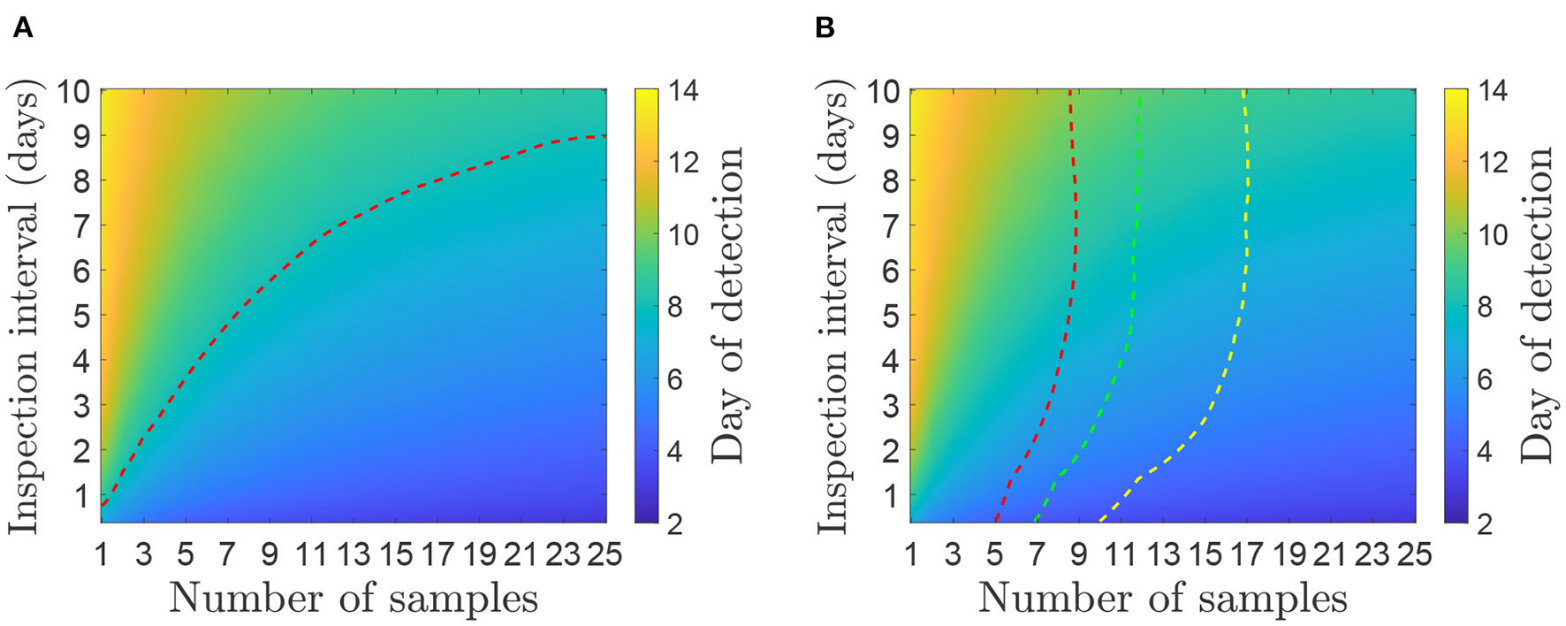
Mean day an outbreak would be first detected under different environmental sampling strategies for a herd size of 50 cattle. **(A)** Comparison with the mean time of detection (8.07 days) estimated for the 2001 UK epidemic (19). **(B)** Comparison with inspection for clinical signs assuming animals are inspected at the same frequency as environmental samples are taken (given on the y-axis). The red dotted line (left) shows day of detection when 5% of the herd are inspected, green line (middle) is 10% and yellow line (right) is 20%. A strategy below or to the right of the dotted line has a lower mean day of detection and therefore performs better than the corresponding level of clinical surveillance.

**Figure 4:**
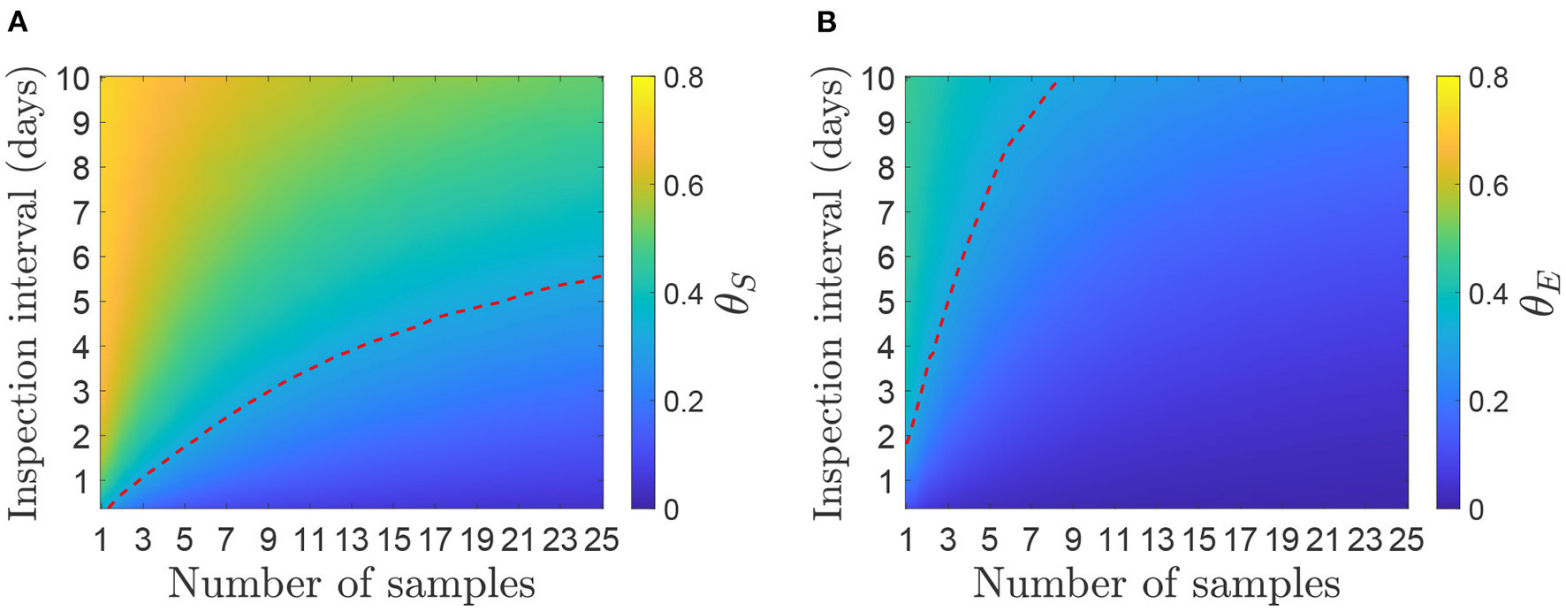
Mean proportion of infectiousness before detection for a herd size of 50 cattle. **(A)** Infectiousness is measured as the sum of viral shedding (θ_*S*_). **(B)** Infectiousness is measured as the sum of environmental contamination (θ_*E*_). The red dotted line represents the level of θ required for *R*_*h*_ = 1 if each measure of infectiousness was the only route of between-herd transmission.

Figures 3, 4 show contour plots for the time to detection, *t*_*d*_, and proportion of infectiousness before detection, θ_*S*_ and θ_*E*_, for different environmental sampling strategies, determined by the number of samples and the number of days between sampling, on a farm with 50 cattle. The two panels in Figure 3 shows the same results, overlaid with different dotted lines for comparison with clinical surveillance strategies. In Figure 3A, the dotted line corresponds to a time detection of 8.07 days, which is the mean estimated for the 2001 UK epidemic (19). This shows the combination of inspection interval and number of samples per inspection required to have the same mean detection time. In Figure 3B, the three dotted lines correspond to the detection time when inspecting cattle for clinical signs. At each inspection interval the dotted line is plotted on the corresponding point on the contour where the mean time of clinical detection is equal to the mean time of detection from environmental sampling.

In both cases, for environmental sampling to be more effective than the alternatives, the number of samples and inspection interval should be chosen to be to the right of or below the dotted lines. For example, a strategy of taking 20 environmental samples every 7 days would detect FMDV after an average of 7 days since infection, which is more effective than inspecting 20% of the herd for clinical signs every 7 days (and would also be more effective than clinical surveillance as implemented in the 2001 UK epidemic). A strategy of taking five samples every 3 days would also take an average of approximately 7 days from infection until detection, but would not be as effective as inspecting 5% of the herd every 3 days. The strategies that perform better than the estimate from the 2001 epidemic and inspections of 20% of the herd require several samples to be taken at a time; options include 12 samples every day, 15 samples every 2 days or 20 samples once a week.

We see from Figure 4 that the proportion of infectiousness before detection is higher for viral shedding than for environmental contamination. This is as expected as the sum of all viral shedding peaks earlier than the environmental contamination, which decays at a slower rate (16). Therefore, at the time of detection, in most scenarios, less than 40% of infectiousness from environmental contamination has occurred compared to up to 70% of that from viral shedding.

Assuming the between-herd reproduction ratio is *R*_*h*_ = 3.2 and infectiousness is measured by either viral shedding or environmental contamination alone to give *R*_*S*_ and *R*_*E*_, respectively, then *R*_*S*_ and *R*_*E*_ = 1 when θ_*S*_ and θ_*E*_ = 0.31, respectively. This is shown by the dotted lines in Figure 4. The area under or to the right of the lines show the required number of samples to be taken to achieve *R*_*h*_ < 1. As θ_*S*_ is higher than θ_*E*_, a frequent sampling strategy is required for *R*_*S*_ < 1, whereas most strategies are below the threshold for *R*_*E*_ < 1. For example, 10 samples once a week would be sufficient to bring θ_*E*_ < 0.31 but not θ_*S*_, whereas 10 samples every 3 days would be sufficient to bring both below the threshold. Strategies of 5 samples every day or 15 every 4 days would also be sufficient for both measures.

The sensitivity of the results on sampling intervals and number of samples taken to changes in herd size was assessed (Supplementary Figures 1–3). This demonstrated that the size of the herd does not have a large impact on θ_*E*_, though smaller herds have a slightly higher detection time and θ_*S*_.

### 3.3. Sampling in an at-risk herd

We now assess the value of environmental sampling in the scenario where a farm is deemed at risk, for example, due to FMDV being detected on a farm nearby or on a farm with close connections.

**Figure 5:**
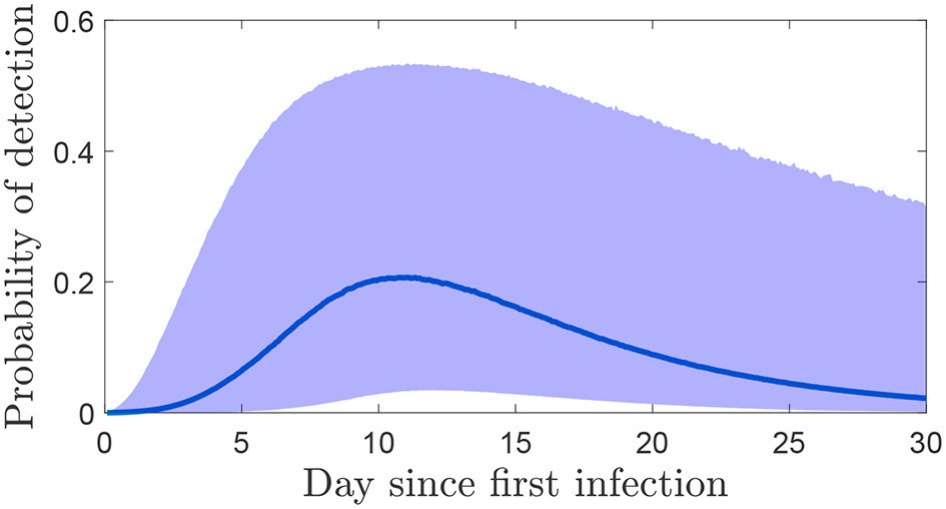
The probability (median and 95% credible interval) of detecting FMDV from a single sample on each day post infection.

The probability of detecting FMDV on an infected farm after a number of days of taking samples is highly dependent on the length of time samples are taken and the time when the first infection occurred. This is shown in the probability of detection in Figure 5 where a single sample is taken from the environment on different days post infection of the herd. The highest probabilities occur at approximately 10 days, so this is when testing should happen to be most confident a premises is not infected.

Sampling in an at-risk herd would start at the time the nearby infected herd was detected. Although in most cases it is unknown for how long the premises could have been infected, we can use the herd generation time (16, 17) to estimate when spread to nearby farms would most likely have occurred. Assuming the detection time distribution follows that given estimated for the 2001 UK epidemic (19) (we could use detection times calculated above, but would have to choose a particular strategy), we can estimate the time since infection that sampling would start. For example, if the herd generation time is 6 days and the detection time is 8 days then sampling on nearby farms would start 2 days after they would most likely have been infected.

**Figure 6:**
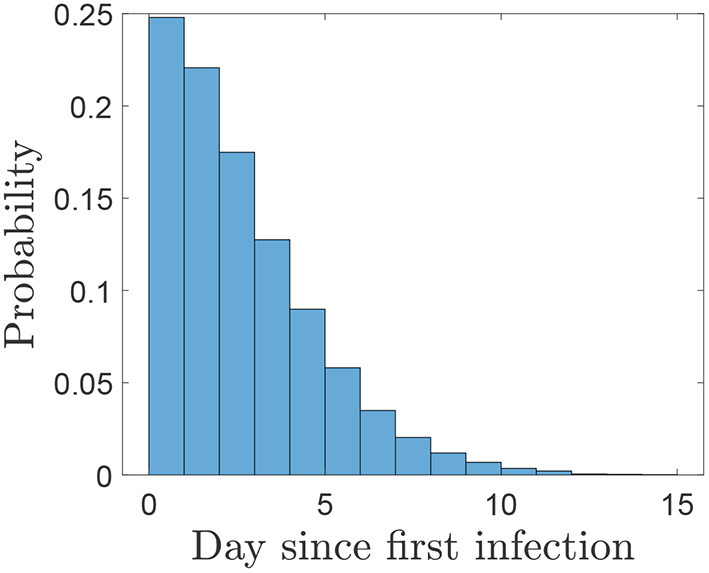
The probability of different time intervals between an infected herd infecting another premises and it being detected. This can be seen as the day that the other premises would be considered at risk and sampling would start. We exclude negative values as they indicate that infection occurs after detection. Estimates for herd generation time are made from viral shedding (*T*_*gd*_) in (16) and detection times from (19).

**Figure 7:**
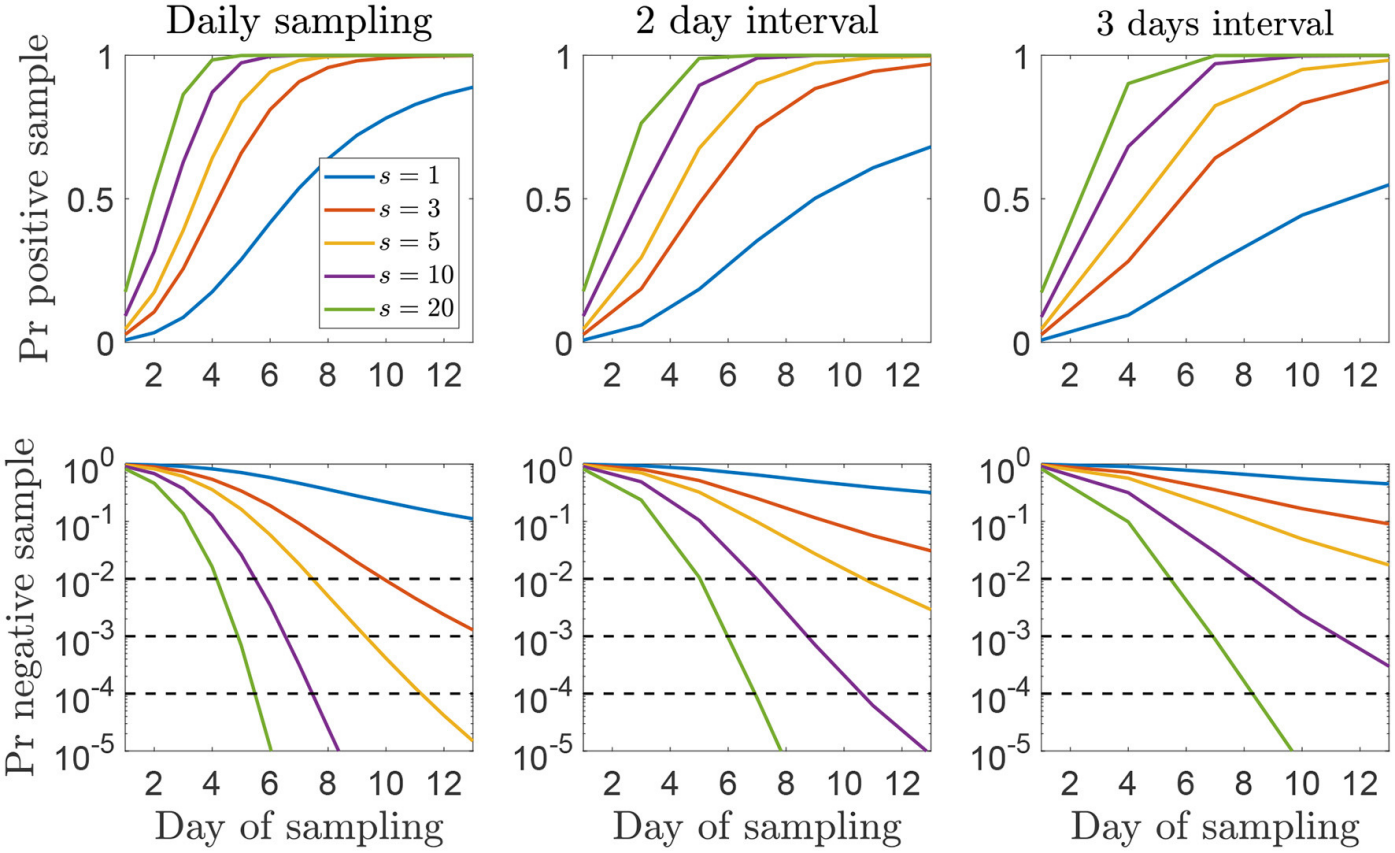
**(Top)** The cumulative probability of detecting FMDV at least once with different sampling strategies on an IP. **(Bottom)** The probability that all samples have tested negative up to and including the day of sampling if a premises is infected. The line color indicates the number of samples taken at each sampling interval, *s* (see legend). The three dotted lines are at 1, 0.1, and 0.01% which correspond to a 99, 99.9, and 99.99% confidence of a negative result. The first sampling day after infection is drawn from the distribution shown in Figure 6.

Using this distribution (Figure 6) the probabilities of different sampling strategies having produced at least one positive sample on, or before, each day of sampling is shown in Figure 7 and the number of days of surveillance required to reach different threshold probabilities of detection using a selection of strategies is given by Table 2. As we would expect, the more samples taken and the more frequently they are taken, the sooner each confidence threshold is reached. Note that when comparing strategies using the same number of samples overall, e.g., 5 samples daily and 10 samples every 2 days, there is little difference in the time to reach each confidence level.

**Table 2:** Median number of days (and 95% credible interval) surveillance is required for to have different % probability of detection where sampling starts on a random day after first infection, given by Figure 6.

**Strategy**	**99%**	**99.9%**	**99.99%**
3 samples daily	10 (5–21)	13 (6–30)	16 (8–36)
5 samples daily	8 (3–15)	10 (4–19)	11 (5–24)
5 samples every 2 days	11 (5–25)	15 (7–33)	17 (9–39)
10 samples every 2 days	9 (3–15)	9 (3–19)	11 (5–25)
10 samples every 3 days	10 (4–19)	13 (4–28)	16 (7–34)
20 samples every 3 days	7 (1–13)	7 (4–16)	10 (4–19)

Similar results are obtained when using environmental contamination instead of total viral shedding as an approximation of between herd infectiousness to calculate the herd generation time [*T*_*ge*_ in (16)]. This is shown in Supplementary Figures 4, 5 where the confidence thresholds are passed at a slightly later time compared to Figure 7.

## 4. Discussion

We have used a previously developed model for the transmission of FMDV through direct contact and environmental transmission (16) to assess the value of environmental sampling as a method of detecting FMDV-infected cattle herds. The probability of detecting FMDV in an environmental sample in the model was parameterized with results from transmission experiments (6).

Samples were taken from four areas of the environment that cattle were kept in: the walls, floor, trough, and feces. The probability of detecting FMDV from a sample was a combination of the probability of virus being present in the precise location sampled, and the sensitivity of the sampling method. We assume that this probability is homogeneous in each of the four locations, although in reality there will likely be areas where more virus accumulates depending on cattle behavior. We also assume a constant viral decay rate, parameterized from the indoor transmission experiments. However, viral decay are likely to be variable and will depend, for example, on environmental factors such as temperature and humidity and the surface material (5, 20, 21).

The probability of detecting FMDV in a single sample is low unless there is a high amount of virus in the environment (see Figure 2). Therefore, a strategy involving taking multiple samples over a period of time is necessary to have a high probability of obtaining a positive sample. In particular, early on in an outbreak there is less virus in the environment and, therefore, either a very large number of samples should be taken, sampling should be continued across several days or both.

The time for detection unsurprisingly increases if the interval between taking samples increases or the number of samples taken decreases. One criterion for judging the effectiveness of a surveillance strategy is if it improves on the mean detection time estimate from 2001 of 8.07 days (19) (see the dotted line in Figure 3A). Such effective strategies include 5 samples every 2 days, 10 samples every 5 days or 20 samples every 8 days. Which choice of strategy in the event of an outbreak will depend on multiple factors including the aims of the surveillance (early detection or proving absence) (22, 23), the cost and availability of sampling and laboratory testing (24, 25), the attitudes of farmers (26) and the wishes of the competent authority, and as such we do not suggest a single ‘best’ strategy.

Detection times using environmental surveillance can be compared with those for clinical inspection as the onset of clinical signs is included in the transmission model. This is illustrated by the dotted lines in Figure 3B, which shows the number of samples needed to improve upon clinical inspection when the inspection interval is the same for both surveillance measures. When the inspection interval is small, fewer samples are required to improve on clinical inspection compared to when the interval is large. For an inspection interval of 5 days or more, the number of samples does not change. This suggests that a good environmental surveillance strategy should prioritize a small inspection interval (i.e., ≤3 days). We note here that if clinical inspection requires the attendance of a dedicated team of veterinarians, this would be a large workload and anyone that attends an IP must isolate for a period of time. Conversely, the environmental sampling method is low-cost, low-technology and could be done by trained individuals or possibly the farmers themselves (7). An economic analysis of various surveillance strategies, such as those conducted in (27, 28), to determine the optimal combination of environmental sampling and clinical inspection would provide additional information for selecting an appropriate strategy, although this is beyond the scope of the present study.

The proportion of infectiousness before detection, θ, can be used to calculate the effective herd reproductive number, *R*_*h*_, when control is applied. In particular, if transmission stops at the time of detection (e.g., because the herd is culled), it shows which strategies will reduce *R*_*h*_ to less than 1, meaning that number of infected herds will decline. This threshold is indicated by the red dotted line in Figure 4, which clearly shows that, for infectiousness from shedding, to achieve *R*_*h*_ < 1 a more demanding strategy is required than one that would match previous detection time estimates (c.f. Figure 3). For infectiousness from environmental contamination, it is easier to achieve *R*_*h*_ < 1 because environmental contamination peaks later than shedding, at approximately 10 days after infection (16). We have treated infectiousness from animal shedding (θ_*S*_) and the environment (θ_*E*_) separately and consider the results as if each were the sole route of between-herd transmission. Although it may be possible to estimate a single θ that incorporates both routes, which would be somewhere between the two results we have shown, it is not clear what the relative contributions of each between-herd transmission route would be. Further investigation into transmission routes between herds would provide this information, but a detailed study of between-herd transmission is outside the scope of this study.

In the cases discussed above, sampling is an ongoing process and there is no particular reason to believe that a premises is infected. In the alternative situation where a herd is deemed at risk of being infected with FMDV, a different strategy will be necessary to either detect FMDV sooner or be confident that transmission did not occur after all (i.e., the herd is free from FMDV). In rare cases it may be possible to identify a particular day in which a herd could have been infected, however usually that is not possible and there is uncertainty in for how long the herd may have been infected. We modeled this uncertainty about the infection day using herd generation times and detection times from past outbreaks. Using this approach, it is far more likely that the infection would have occurred recently, 65% within the last 3 days and 86% within the last 5 days. This means that confidence that a series of negative results indicates a herd is free from infection takes longer to achieve as early in an outbreak there is less virus accumulated in the environment. If an infection occurred 10 days ago, there is a much higher probability of detecting it immediately than if it arrived 3 days ago (Figure 5). While it is clear that a single-sample strategy is never sufficient, the choice of the number of samples that should be taken and how frequently, depends on the required confidence level and how quickly it should be arrived at. However, because of the time taken for virus to accumulate at the start of an outbreak, a reasonable confidence level in less than 1 week is unlikely to be achieved.

Here we have compared detection from environmental surveillance with clinical inspection. However, FMDV can also be detected in blood, nasal fluid and saliva and surveillance based on these types of sample has previously been investigated by Nelson et al. (18). In particular, they determined the reduction in the between-herd reproduction ratio *R*_*h*_ through surveillance *via* different sampling strategies [see Table 2 in (18)], which can be compared to the proportion of infectiousness, θ_*S*_ and θ_*E*_, shown in Figure 4. For example, they found that taking nasal or saliva swabs from 5 animals once a week would reduce *R*_*h*_ from 3.2 to 0.8 which is an equivalent of θ = 0.25. The same would be achieved by 8 environmental samples every 2 days if we use θ_*S*_ to represent infectiousness, or 8 environmental samples once a week using θ_*E*_. This suggests that animal sampling is the more efficient approach, although the low cost and ease of use of environmental samples may make environmental surveillance more efficient during an epidemic, where trained professionals required to take animal swabs will be in high demand. Also note that they used a different model which may affect the infectiousness profile and, hence, conclusions about the reduction in transmission for the different surveillance strategies.

Our results demonstrate that environmental sampling is a potentially useful tool to use during a FMD outbreak. Environmental sampling has previously been shown to successfully detect FMDV in countries where FMD is endemic (7, 8). Here we have shown that it could play a role in FMD-free countries too, where the aim is to eradicate the disease through early detection. If a suitable strategy is used, environmental sampling can produce detection times much lower than during the 2001 UK outbreak. It is also a low-cost and easy to use sampling method that can reduce the demand on trained veterinarians. Approximately 6.5 million animals were culled in the UK during the 2001 outbreak, in part due to a policy of culling at-risk farms (4). If careful surveillance strategies are applied, such as the ones described in this paper, it could reduce the need for culling and detect subsequent outbreaks quickly. Sampling to prove absence of FMDV could also be used as part of a wider surveillance strategy, such as discussed by (22), to regain an FMDV-free status.

The methodology behind this work and the previously developed model (16) is adaptable and could be used for other pathogens that are detectable in the environment as well as examining other locations, such as markets, or including other livestock. It could be useful to consider sheep in particular as it is often difficult to detect FMDV based on clinical signs in this species (1). Although the virus decay rate and the detection probability would be the same, data would need to be collected to parameterize the virus accumulation rate from sheep and develop an accurate transmission model.

## Data availability statement

The datasets presented in this study can be found in online repositories. The names of the repository/repositories and accession number(s) can be found below: https://github.com/DrJREllis/FMDV_Sampling_Model.

## Author contributions

SG conceived the study. SG and JE designed the study. CC and EB provided data. JE implemented the model and wrote the first draft of the manuscript. All authors contributed to manuscript revision, read, and approved the submitted version.
