# Supplementary material for: Assessing the effectiveness of environmental sampling for surveillance of foot-and-mouth disease virus in a cattle herd
Source: Front Vet Sci. 2023 Mar 13;10:1074264. doi: 10.3389/fvets.2023.1074264 (PMC10040685; doi:10.3389/fvets.2023.1074264)
Supplement: Supplementary file 1 [file Data_Sheet_1.PDF]

Supplementary Material

1 HERD SIZE COMPARISONS

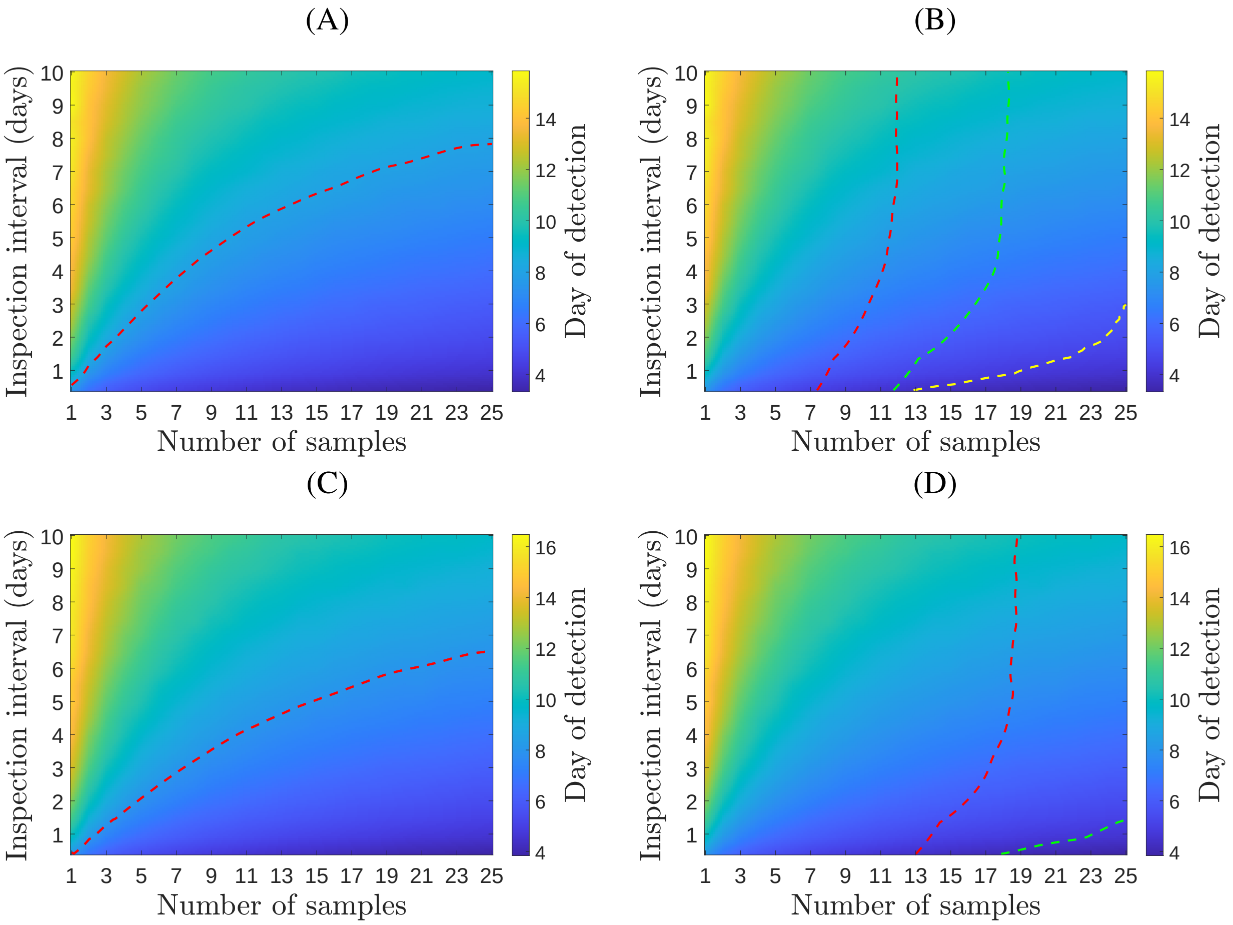

Figure S1. The mean day the outbreak would be first detected under different sampling strategies for a herd size of (A,B) 100 and (C,D) 200 cattle. (A,C) The dotted line represents the mean day of detection according to [2] of 8.07 days. (B,D) Dotted lines represent the equivalent day of detection from inspecting for clinical signs assuming inspection intervals at the same frequency as environmental samples are taken (given on the y-axis). Red line (left) shows day of detection when 5% of the herd are inspected, green line (middle) is 10% and yellow line (right) is 20%. A surveillance strategy below or to the right of the dotted line has a lower mean day of detection and therefore performs better.

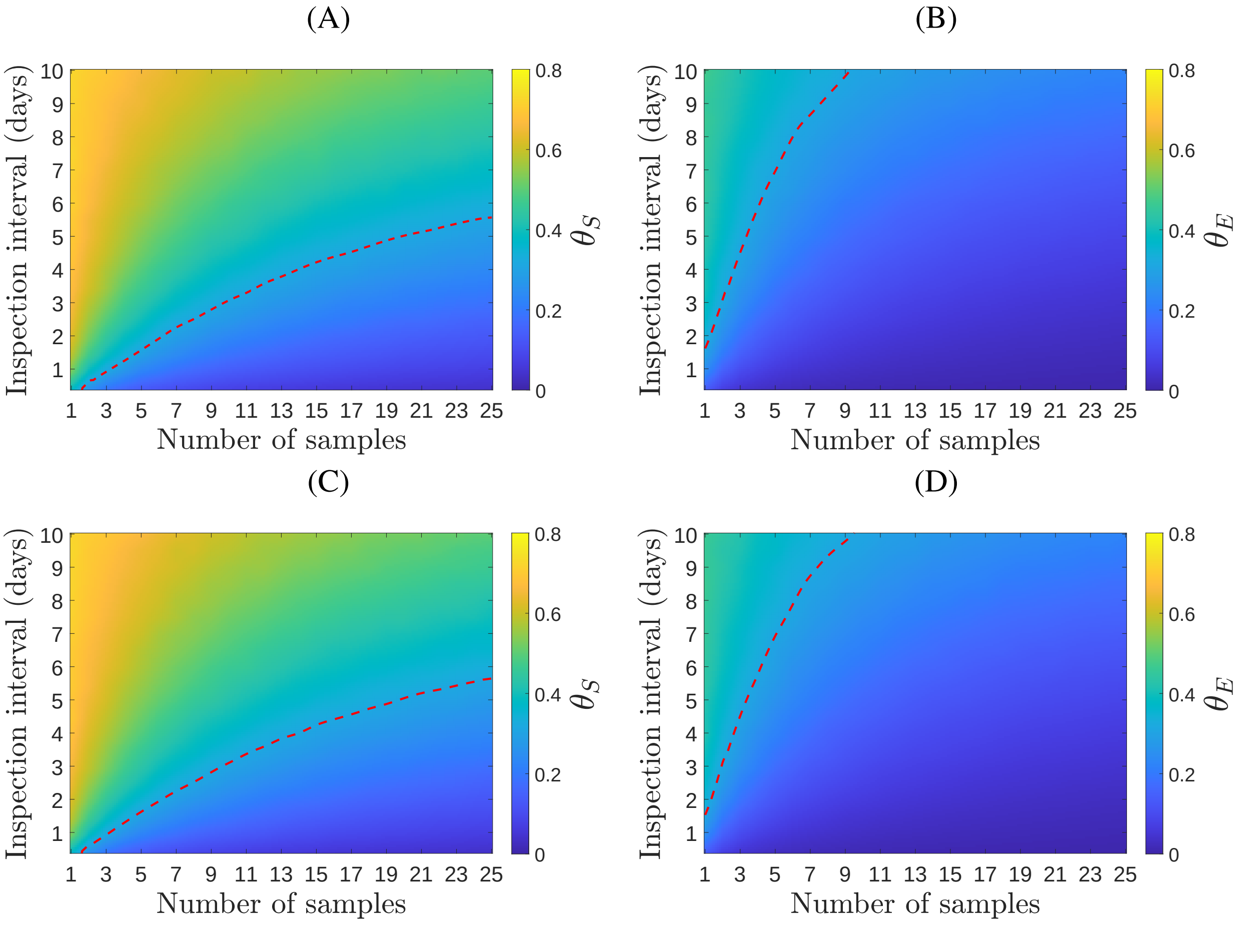

Figure S2. The mean proportion of infectiousness before detection for a herd size of (A,B) 100 and (C,D) 200 cattle. (A,C) Infectiousness is measured as the sum of viral shedding (θ_S). (B,D) Infectiousness is measured as the sum of environmental contamination (θ_E). Dotted lines correspond to $\theta = 0.31$.

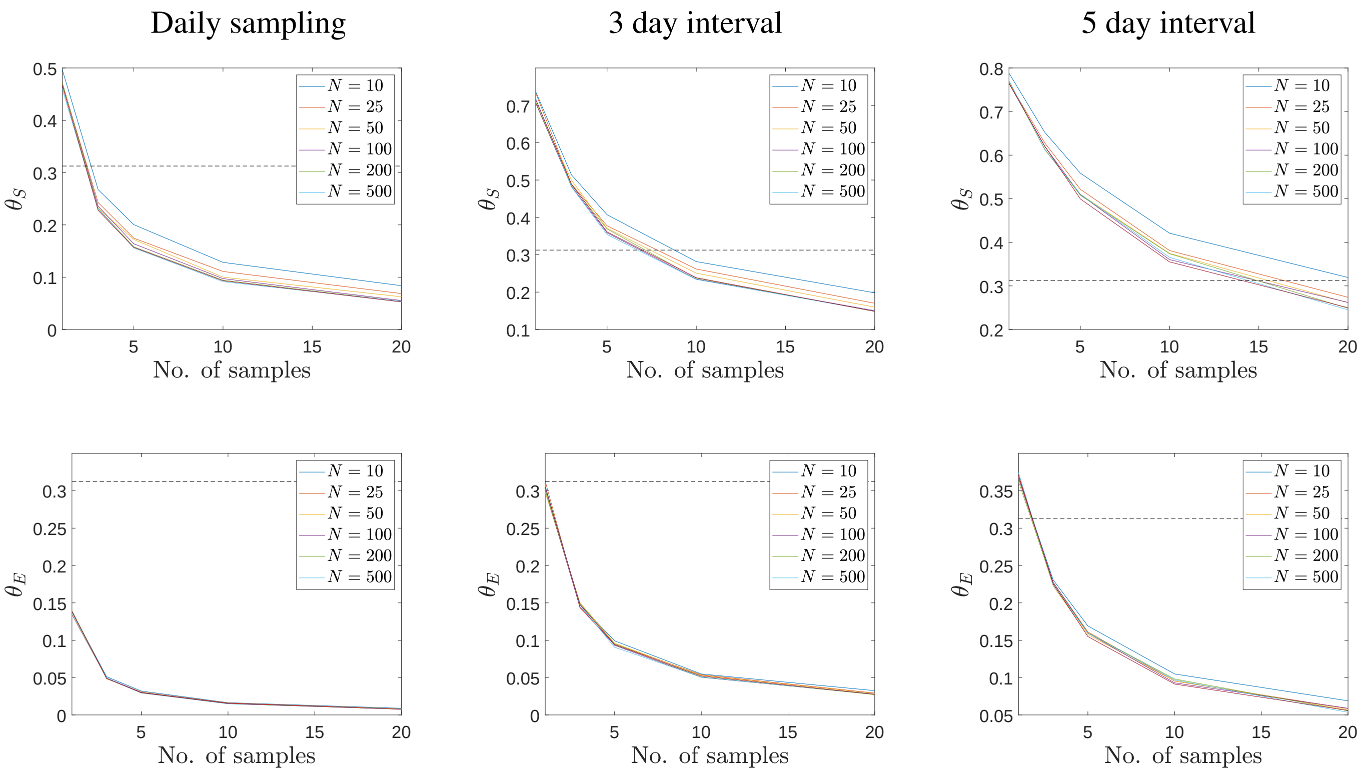

Figure S3. Proportion of infection for direct contact (top row) and environmental contamination (bottom row) that occurs before detection with different sampling intervals (columns), herd sizes (coloured lines) and number of samples taken per interval (x axis). Dotted line represents the value of θ that would make $R_h = 1$.

2 SAMPLING IN AN AT-RISK HERD

In Fig. 6, we use the herd generation time calculated using the viral shedding results obtained by the model described in [1]. This would assume that viral shedding is responsible for transmission, perhaps via the movement of animals. If transmission occurs from the movement of fomites, environmental contamination will be a more appropriate measure to calculate the herd generation time.

As shown in Fig. S4, there is a small probability that infection from environmental contamination will occur before detection. If it does, then it is likely to have been more recent when compared to infection from viral shedding, e.g. the probability of infection happening up to one day before sampling is approximately 0.45 compared to 0.25 in Fig. 6. This means that the probability of detection increases slightly slower in Fig. S5 than in Fig. 7, as recent infections take longer to be detected because of the time taken for virus to accumulate in the environment.

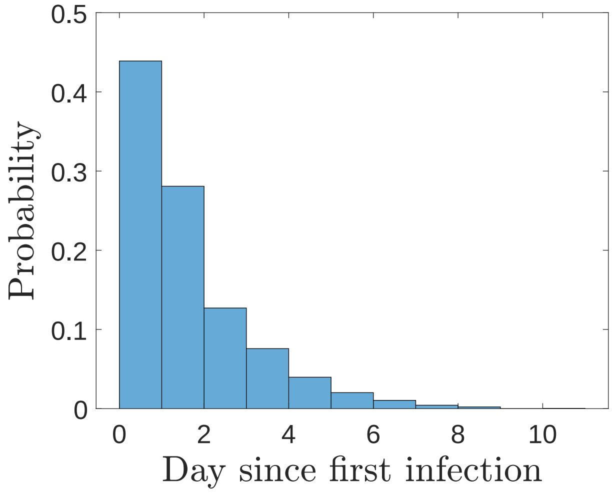

Figure S4. The probability of different time intervals between an infected farm infecting another premises and it being detected. This can be seen as the day that the other premises would be considered at risk and sampling would start. (A) All values, (b) excluding negative values. In reality, negative numbers are unlikely as they indicate that infection occurs after detection. Estimates for herd generation time are made from environmental contamination (T_{ge}) in [1] and detection times from [2].

3 CODE AVAILABILITY

All code and data used for this model are available online at
`https://github.com/DrJREllis/Inferring-transmission-routes-FMDV`

REFERENCES

[1]J. Ellis, E. Brown, C. Colenutt, D. Schley, and S. Gubbins. Inferring transmission routes for foot-and-mouth disease virus within a cattle herd using approximate Bayesian computation. *BioRxiv*, 2022.

[2]I. C. Ster, B. K. Singh, and N. M. Ferguson. Epidemiological inference for partially observed epidemics: the example of the 2001 foot and mouth epidemic in Great Britain. *Epidemics*, 1(1):21–34, 2009.

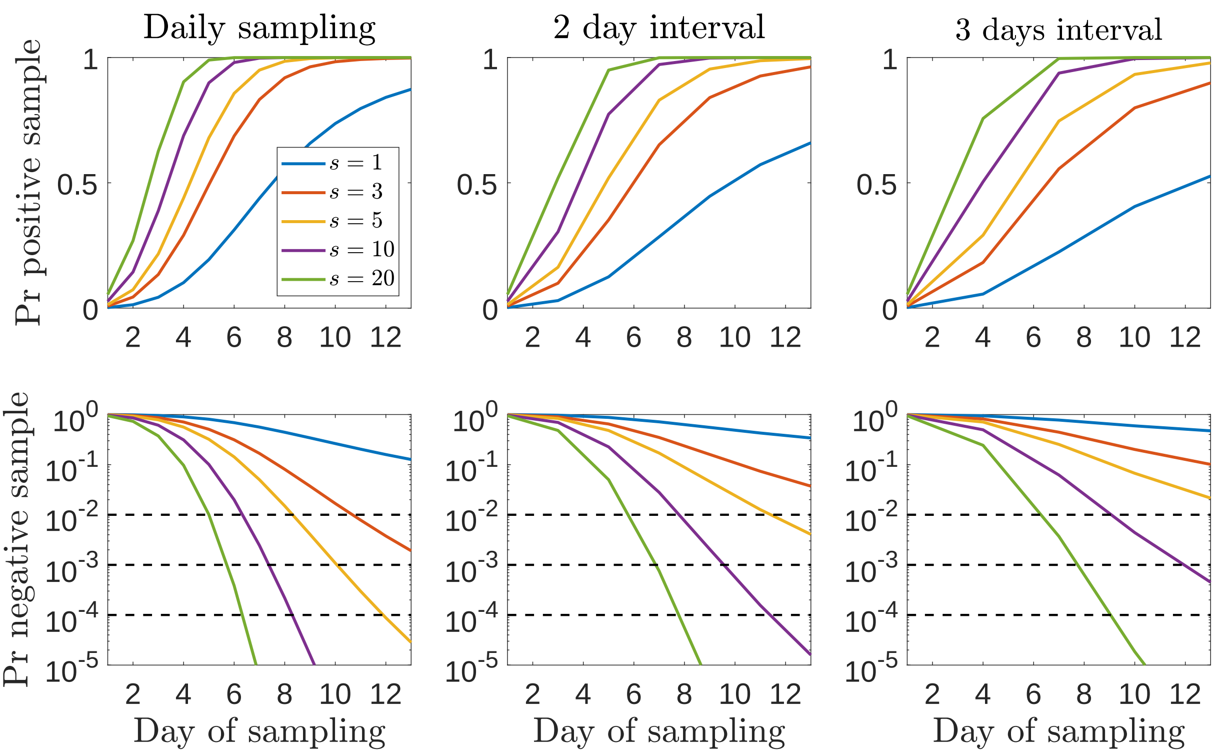

Figure S5. (Top) The cumulative probability of detecting FMDV at least once with different sampling strategies on an IP. (Bottom) The probability that all samples have tested negative up to and including the day of sampling if a premises is infected. s is the number of samples taken at each sampling interval. The three dotted lines are at 1%, 0.1% and 0.01% which correspond to a 99%, 99.9% and 99.99% confidence of a negative sample. The first sampling day after infection is drawn from the distribution shown in Fig. S4.
